# Development of a human primary gut-on-a-chip to model inflammatory processes

**DOI:** 10.1038/s41598-020-78359-2

**Published:** 2020-12-08

**Authors:** Claudia Beaurivage, Auste Kanapeckaite, Cindy Loomans, Kai S. Erdmann, Jan Stallen, Richard A. J. Janssen

**Affiliations:** 1 Galapagos BV, Leiden, South Holland 2333CL The Netherlands; 2 Department of Biomedical Science, Faculty of Science, University of Sheffield, Sheffield, S10 2TN South Yorkshire UK

**Keywords:** Drug discovery, Cytokines, Autoimmunity, Inflammatory bowel disease

## Abstract

Inflammatory bowel disease (IBD) is a complex multi-factorial disease for which physiologically relevant in vitro models are lacking. Existing models are often a compromise between biological relevance and scalability. Here, we integrated intestinal epithelial cells (IEC) derived from human intestinal organoids with monocyte-derived macrophages, in a gut-on-a-chip platform to model the human intestine and key aspects of IBD. The microfluidic culture of IEC lead to an increased polarization and differentiation state that closely resembled the expression profile of human colon in vivo. Activation of the model resulted in the polarized secretion of CXCL10, IL-8 and CCL-20 by IEC and could efficiently be prevented by TPCA-1 exposure. Importantly, upregulated gene expression by the inflammatory trigger correlated with dysregulated pathways in IBD patients. Finally, integration of activated macrophages offers a first-step towards a multi-factorial amenable IBD platform that could be scaled up to assess compound efficacy at early stages of drug development or in personalized medicine.

## Introduction

Inflammatory bowel disease (IBD), including Crohn’s disease and ulcerative colitis, is a group of chronic relapsing inflammatory conditions of the gastrointestinal tract. Their pathogenesis is not fully understood yet. Nevertheless, they are thought to arise in genetically susceptible individuals in response to several factors such as diet, microbiota, smoking or environmental factors^1,2^. Mechanistically, IBD patients may harbour genetic mutations in genes involved in the integrity of the intestinal epithelium, which allows microbiota to translocate in the intestinal tissue and initiate an inflammatory reaction leading to intestinal lesions showing excessive immune cell infiltration^3^. Immune cells recruited to the intestinal mucosa are generally sufficient to dial down inflammation and return the microenvironment to a normal status. However, IBD patients lack the required regulatory mechanisms or have aberrant activation of these immune cells, leading to a sustained inflammatory state^1,3^. Notably, the intestinal tissue of actively inflamed IBD patients show a drastic increase of activated macrophages; the number of pro-inflammatory M1 macrophages will increase as the number of anti-inflammatory M2 macrophages will decrease^4–6^. Obviously, macrophages are not the only cell type playing a role in the pathogenesis of IBD; dendritic cells^7^, innate lymphoid cells^8^ as well as T helper cells^6^ have also been shown to be involved or disbalanced through the disease progression.

Trying to understand the mechanisms behind the establishment of these diseases is particularly challenging, as modeling multi-factorial diseases in an in vitro environment is difficult. Current in vitro IBD models mostly rely on adenocarcinoma-derived Caco-2 cells^9,10^. Caco-2 cells have provided great insight in the importance of the epithelial barrier function in intestinal inflammation^11,12^, however they do not represent the cellular diversity of the intestinal epithelium and therefore poorly reflect in vivo physiology. Furthermore, they fail to express some of the major inflammatory cytokines involved in IBD upon stimulation^13,14^. On the other hand, human primary intestinal epithelial cells (IEC) are a physiologically relevant option that can easily be cultured as human intestinal organoids (HIO). HIO contain multiple epithelial cell types derived from the intestinal stem cells and therefore faithfully recapitulate the cellular diversity of the intestinal epithelium in vivo^15,16^. However, one major drawback of HIO is their enclosed lumen because of the spherical nature of organoids, making luminal drug exposure difficult. Additionally, HIO cultures lack tissue-tissue interactions, mechanical tension, as well as the integration of other cell types important to intestinal physiology such as immune and vascular cells. Thus, there is a major need for more complex and physiologically relevant intestinal organ culture systems.

Recently, the organ-on-a-chip technology has emerged as an alternative approach that offers different perfusable microchannels in which cell types of interest can be cultured together in an organ-like orientation using the support of an extracellular matrix (ECM) gel^17,18^. A few gut-on-a-chip models have already been developed with adenocarcinoma-derived cell lines such as Caco-2 cells and HT-29^12,19–22^. Because of the limited physiological relevance of these intestinal cell lines, a few studies have created gut-on-a-chip models using HIO or iPSC-derived organoids^18,23–25^. These studies clearly showed the augmented relevance of human primary models over Caco-2 cells and even established co-cultures with microvascular endothelial cells and intestinal myofibroblasts^23,24^. Importantly, the use of organ-on-a-chip technology transcends the limitation of ‘inside-out’ HIO as it allows the cells to grow in a monolayer that will cover all the surfaces of the microfluidic channel thus creating a tubular structure with an open lumen, allowing exposure to factors such as bacteria, cytokines or drugs in a localized manner. Despite the remarkable advance that these results represent, no study has yet applied their human primary gut-on-a-chip device for the recapitulation and investigation of disease-specific phenotypic characteristics of IBD. Furthermore, the lack of efficient treatment for IBD patients urges scientists to develop devices and models that allow screening of compounds in a relatively high-throughput manner. The commonly used PDMS-based chips lack the necessary scalability to perform such high throughput experiments and lead to absorption of small molecule drugs^26,27^, making these inadequate for drug screening.

Herein, we report the establishment of a gut-on-a-chip model composed exclusively of patient-derived human primary material. Monocyte-derived macrophages and HIO from different donors were integrated in the membrane-free 3-lane OrganoPlate platform, a higher throughput alternative to PDMS-based chips that would allow compound screening due to its glass composition. We show that the microfluidic culture of HIO leads to an increased polarization and differentiation profile of the cells over standard organoids culture. Importantly, the transcriptome of the microfluidic gut-on-chip resembles that of adult human normal colon in vivo. We induced IBD hallmarks by triggering the gut-on-a-chip with lipopolysaccharide (LPS) and interferon-gamma (IFN-γ), leading to the activation and increase cytokine production of both HIO and macrophages. Our model could individually pinpoint cell type-specific cytokine production, in both apical and basal channels simultaneously, allowing us to fully characterize the induction of the inflammatory phenotype. Induction of inflammation in HIO was also confirmed by transcriptomics of the microfluidic gut-on-a-chip. Finally, the suitability of the model to perform compound exposure experiments was confirmed. Our proof-of-concept experiments show that known anti-inflammatory compound TPCA-1 prevents the establishment of the inflammatory phenotype in a dose-dependent manner. In summary, our results highlight the suitability of our in vitro microfluidic gut-on-a-chip model to mimic key physiological aspects of the intestine and further apply them to IBD modelling and potentially large-scale screening in the search for novel IBD treatments.

## Results

### Primary human gut-on-a-chip developed using biopsy-derived organoids

**Figure 1:**
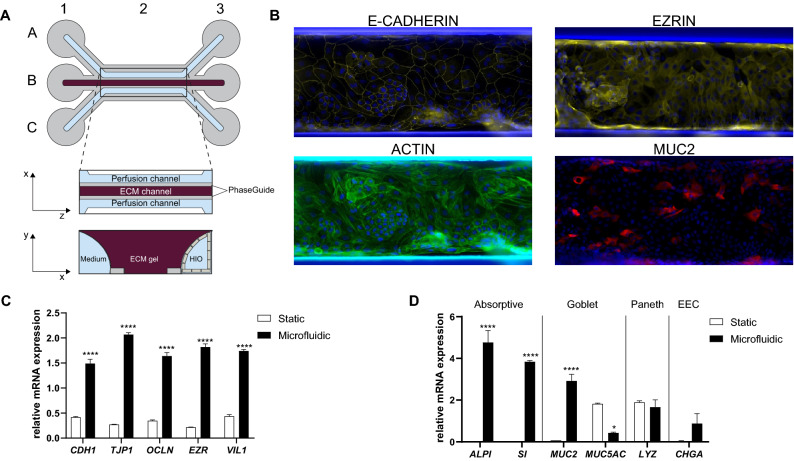
IEC cultured under microfluidic conditions show higher expression of polarization and differentiation markers. (**A**) Schematic representation of the model. One microfluidic chip is composed of three channels: the upper channel (**A**), middle channel (**B**) and lower channel (**C**). Each channel has dedicated inlets (A1, B1, C1) and outlets (A3, B3, C3). Imaging is possible through the observation window (B2). The top view shows the two perfusion channels flanking the middle channel where an extracellular matrix (ECM) gel is seeded. The PhaseGuides allow the ECM gel to form a meniscus, allowing barrier-free culture. The transversal view shows the IEC forming an epithelial tubule in the upper channel against the ECM gel. Medium perfusion is started by placing the OrganoPlate on an interval rocker. Illustration created with Adobe Illustrator software (version22.1, https://adobe.com/products/illustrator). (**B**) Representative 20X stitched photographs of *zonula adherens* marker E-CADHERIN, apical polarization marker EZRIN, polarization marker ACTIN and goblet cell marker MUC2 in HIO grown in microfluidic conditions for 10 days. Blue depicts nuclei by DAPI staining. Images were created with the Image J software (version 1.47v, https://imagej.nih.gov/ij/). (**C**,**D**) mRNA expression level of polarization markers (**C**) *CDH1*, *TJP1*, *OCLN*, *EZR* and *VIL1* and differentiation markers (**D**) *ALPI*, *SI*, *MUC2*, *MUC5AC*, *LYZ* and *CHGA* in HIO donor 2. HIO were grown in standard static culture or in monolayers subjected to fluid flow (microfluidic) for 8 days. Graph shows average relative expression values normalized to *RPS18*, *HPRT1*, *GUSB* and *YWHAZ* ± SEM with unpaired t-test compared to standard HIO culture (n = 3).

Presently, the unique source for primary intestinal cells are organoid cultures derived from patients’ biopsies. We set out to develop a gut-on-a-chip model integrating human biopsy-derived epithelium under fluid flow to determine if such a model would better reflect the in vivo situation. HIO cultures were established using intestinal crypts derived from macroscopically normal regions of intestinal endoscopic biopsies (Table 1). After stabilization and expansion of the cultures, HIO were dissociated and seeded in the upper channel of the 3-lane OrganoPlate, on top of an ECM gel contained in the middle channel (Fig. 1A).

**Table 1:** HIO donor information.

HIO donor	Biopsy location	Gender donor	Age donor (years old)	Supplier
1	Rectum	Female	9	Hubrecht Institute
2	Colon	Male	72	Tissue Solutions
3	Sigmoid colon	Male	50	Tissue Solutions

The composition of the human intestinal stem cell (HISC) medium previously published by Hans Clevers’ group^28^ (Table 2) was optimized to favour IEC attachment in the OrganoPlate and ALK5 inhibitor A83-01 was withdrawn from the formulation (attachment medium). IEC kept in attachment medium for the full duration of the experiment started to become mesenchymal as indicated by the upregulation of SM22 expression (Fig. S1). By switching back to HISC medium two days after seeding, IEC did not gain mesenchymal markers expression but did form confluent epithelial monolayers in a more robust and reproducible way (Fig. S1).The IEC first adhered to the ECM gel before they could cover all the walls of the upper microfluidic channel, therefore forming a polarized epithelial tubule, as previously reported in similar gut-on-a-chip models^19,29^.

**Table 2:** Composition of the HISC medium.

Constituent	Supplier (Catalog number)	Final concentration
rh R-spondin 3	R&D systems (3500-RS/CF)	0.5 µg/mL
rh Noggin	PeproTech (120-10C)	0.1 µg/mL
B27 supplement	Gibco (17504)	1X
N-Acetyl-L-cysteine	Sigma Aldrich (A9165)	1.25 mM
Nicotinamide	Sigma Aldrich (N0636)	0.01 M
rh EGF	PeproTech (AF-100-15)	0.05 µg/mL
rh Gastrin 1	Tocris (3006)	0.005 µM
Primocin	InvivoGen (ant-pm)	0.1 mg/mL
A83-01	Tocris (2939)	0.5 µM
SB202190	Sigma Aldrich (S7067)	0.01 mM
Y-27632	Abmole (M1817)	0.01 mM
Wnt3a conditioned medium	-	50%

After 10 days of microfluidic culture, the polarization status of the IEC was assessed (Fig. 1B). The localization of E-CADHERIN at cell–cell junctions indicated the formation of *zonula adherens* junctions. Similarly, EZRIN expression was present and its localization was restricted to the apical surface of IEC, confirming proper polarization of the cells. IEC showed unified organization of ACTIN filaments further supporting the conserved apical-basal polarity of the IEC. The expression level of polarization markers was also confirmed at the mRNA level by qPCR by comparing HIO grown in standard Matrigel droplets (static) and IEC grown in an Organoplate subjected to fluid flow (microfluidic). The microfluidic culture of HIO increased the expression of polarization markers *CDH1*, *TJP1*, *OCLN*, *EZR* and *VIL1* when compared to static HIO culture (Fig. 1C). These results were confirmed in additional HIO donor (Fig. S2A).

We further assessed the differentiation state of the IEC in the microfluidic chip. The presence of enterocytes, the predominant cell type of the intestinal epithelium, was confirmed by the successful mRNA detection of the brush border enzymes *ALPI* and *SI* (Fig. 1D). The presence of goblet cells, was highlighted by a MUC2 staining of IEC grown under fluid flow for 10 days (Fig. 1B) and the mRNA levels of *MUC2* and *MUC5AC* expression (Fig. 1D). The microfluidic culture of IEC led to an increase of the expression of these markers at the mRNA level compared to the static HIO culture. As the cells used in this study are derived from distal colonic and rectal portions of the gastrointestinal tract (Table 1), limited expression of Paneth cell marker *LYZ* and enteroendocrine cell marker *CHGA* could be detected and was not significantly upregulated upon microfluidic culture of the cells (Fig. 1D). These results were confirmed in additional HIO donor (Fig. S2B). Furthermore, the microfluidic culture seemed to increase the general differentiation state of IEC at the expense of proliferation as noted by the upregulation of expression of transcription factors involved in differentiation programs *HES1*, *CDX2* and *KLF4* and a decrease of expression of stem cell marker *LGR5* (Fig. S3A,B). Interestingly, we found stem cell marker *OLFM4* expression to be upregulated in microfluidic-grown IEC (Fig. S3C). Overall, these results indicate that IEC can be grown under microfluidic conditions and that fluid flow increases the expression of polarization and differentiation markers.

### Transcriptomic comparison of the microfluidic gut-on-a-chip versus standard HIO culture

**Figure 2:**
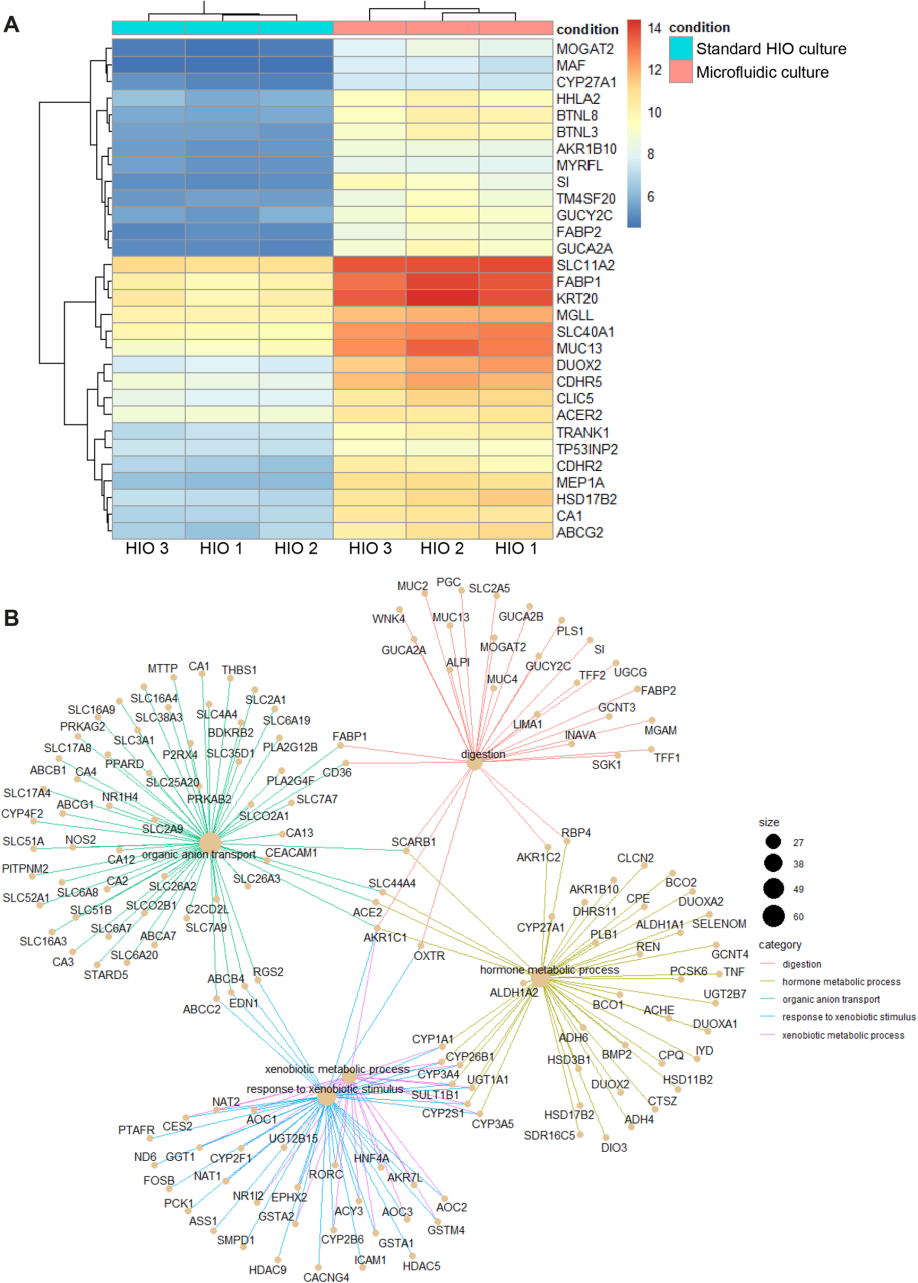
IEC cultured under microfluidic conditions show higher expression of genes involved in digestion, intestinal transport and hormone metabolic process. (**A**) Heatmap representation of the top 30 differentially expressed genes between standard and microfluidic HIO culture in three HIO donors. Higher levels of expression are represented in red and lower levels in blue. (**B**) Differentially-expressed genes between standard and microfluidic HIO culture were grouped under Gene ontology (GO) functional pathways. Circle size represents the number of genes differentially expressed in each GO pathway and lines link individual genes to their related GO pathways.

To further determine whether the microfluidic culture of IEC affects the expression of physiologically relevant genes, we carried out transcriptome-wide analysis of IEC from three different donors. We performed a head-to-head transcriptomic comparison of the established gut-on-chip cultures with the original HIO cultures used for chip seeding. A non-biased analysis of all 4,921 significantly up- and down-regulated genes was performed and the top 30 differentially expressed genes in microfluidic vs standard HIO culture conditions is shown at Fig. 2A (PCA graph can be found at Fig. S4A). In order to uncover the presence of physiologically relevant pathways, Gene Ontology (GO) enrichment analysis for all differentially expressed genes was executed. Interestingly, the analysis uncovered that gene sets involved in digestion, intestinal transport and hormone metabolic process were differentially expressed between microfluidic culture and static cultures (Fig. 2B). These results were confirmed by qPCR, where the microfluidic culture increased the expression of intestinal enzymes *SI* and *ALPI* (Fig. 1D).

**Figure 3:**
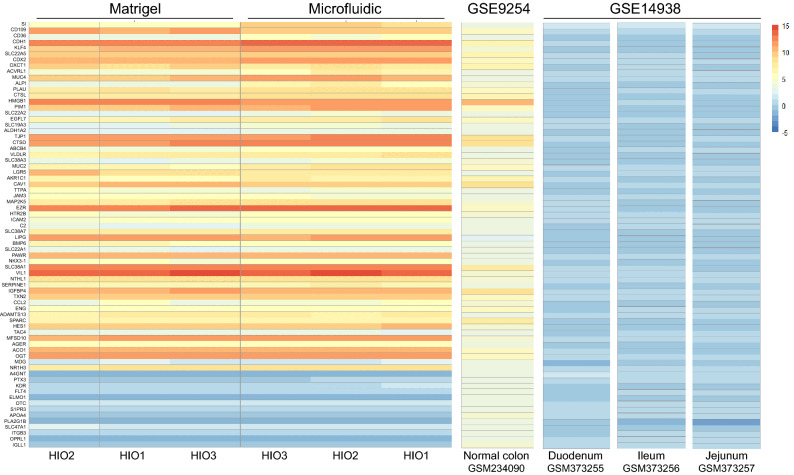
RNA expression profiles of HIO donors resemble human colonic mucosa. Heatmap representation of the RNA expression profile of 72 genes defining colonic identity in 3 HIO donors grown in microfluidic or standard HIO culture conditions for 8 days, compared with human normal colon from study GSE9254^31^ and human normal small intestine sections (duodenum, jejunum, ileum) from study GSE14938^30^. Genes with higher levels of expression are represented in red and genes with lower levels of expression in blue.

Additionally, we compared the samples’ transcriptomic results with gene profiles of distinct regions of the human gastrointestinal tract (ileum, jejunum, duodenum and colon)^30,31^. As the organoids cultures were derived from distal colon biopsies (Table 1), a panel of genes defining colonic tissue was selected for this purpose. Within this panel of genes, only subtle variations between static and microfluidic samples could be observed. Overall, the profiles resembled those of fresh human normal colon and the microfluidic culture did not alter tissue phenotype (Fig. 3). We also compared our datasets to a Caco-2 gut-on-a-chip and Transwell models published previously^24^ and the differences were drastic; Caco-2 cells had lower expression for all tested genes and were more similar to small intestine tissue sections (Fig. S5). Once again, the microfluidic culture of Caco-2 cells did not have a striking effect on the expression of this specific set of genes over Transwell static culture. In conclusion, it appears that HIO have a higher expression of genes involved in intestinal transport and digestion pathways when they are cultured under microfluidic conditions, while their overall colonic tissue phenotype is preserved.

### Activation of the gut-on-a-chip towards an inflammatory state

Following the establishment of a physiologically relevant gut-on-a-chip, we wanted to determine whether our model could recapitulate main characteristics of intestinal inflammation. To do so, the microfluidic gut-on-a-chip was exposed to LPS and IFN-γ. IFN-γ is one of the most highly upregulated cytokines after microbial invasion and in chronic inflammatory diseases including IBD^1,32^. It is produced by innate lymphoid cells and has been shown to selectively alter the permeability of the intestinal epithelium, allowing the translocation of bacterial components to the intestinal tissue^33–35^. We applied the inflammatory trigger on both the apical and basolateral side in order to promote the complete activation and differentiation of macrophages into M1 macrophages, work that will be presented in the last section of this report. However, we did control asymmetric responses to the inflammatory stimuli in HIO (Fig. S6). Overall, there were no differences on cytokine production whether the LPS was applied apically or basally with IFN-γ kept on the basal side only. However, when LPS and IFN-γ were simultaneously applied on both apical and basal sides, the apical release of cytokines IL-6 and GM-CSF were increased, while IL12-p70 remained constant and cytokines IL-1β and CXCL10 were decreased (Fig. S6).

**Figure 4:**
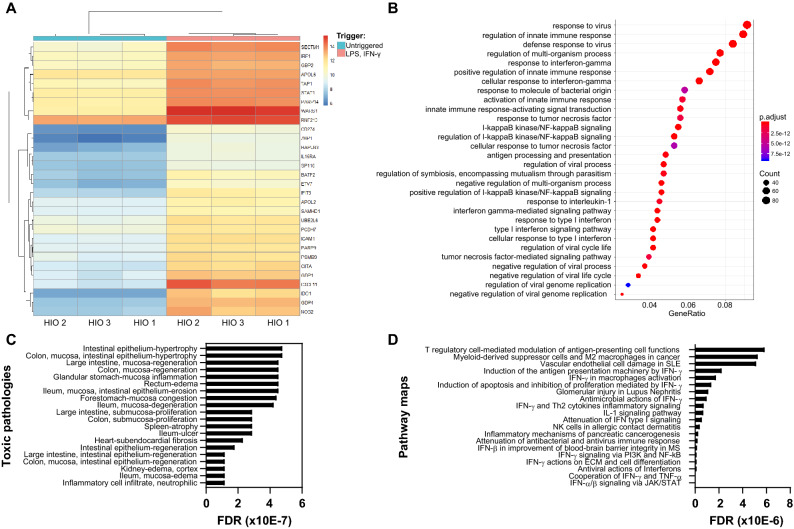
Induction of inflammatory conditions upregulates pathways involved in cytokine and immune signaling in IEC. (**A**) Heatmap representation of the top 30 differentially expressed genes between normal (untriggered) and inflammatory conditions (LPS 100 ng/mL, IFN-γ 20 ng/mL) in 3 different HIO donors. IEC were grown under microfluidic conditions for 8 days and triggered for 24 h. Heatmap shows gene expression levels (adjusted p value). Genes with higher expression values are shown in red and genes with lower expressions values in blue. (**B**) Based on significantly changed gene sets between normal and inflammatory conditions, functional Gene ontology (GO) pathways were isolated. Circle size represents gene counts and color gradation represents adjusted p values. (**C,D**) The top 30 differentially expressed genes were queried against toxic pathologies (**C**) and pathways (**D**) in Metacore. Graphs show false discovery rate (FDR) values.

The induction of inflammatory conditions in the gut-on-a-chip was first confirmed by RNA-sequencing of non-triggered and triggered samples from three HIO donors cultured in microfluidic conditions. A non-biased analysis of all 1,807 significantly up- and down-regulated genes was performed and the top 30 differentially expressed genes in triggered vs untriggered HIO grown in microfluidic conditions is shown at Fig. 4A (PCA graph can be found at Fig. S4B). All genes differentially expressed between the two experimental groups were assembled into functional GO pathways. When the gut-on-chip model was triggered with of LPS and IFN-γ, pathways involved in the regulation of IFN-γ/TNF-α/IL-1 responses, in the activation of the innate immune response and in the response to bacterial factors were over represented compared to the non-triggered condition (Fig. 4B). We used Metacore to map the top 30 differentially expressed genes against available disease maps and observed that they correlated strongly with IBD (p-value 7.402E-6, FDR 5.796E-5) and, to a lesser extent, with gastrointestinal diseases in general (p-value 1.002E-2, FDR 1.917E-2). All top scoring pathologies did relate to the gastrointestinal tract or inflammation (Fig. 4C) and more detailed analysis of the perturbed pathways further confirmed that the gene set identified in the triggered cultures is involved in immune regulation at many different levels (Fig. 4D). These results confirmed that the trigger, composed of LPS and IFN-γ, successfully induced a pro-inflammatory state in the IEC and support the relevance of the model.

### Anti-inflammatory compound exposure

**Figure 5:**
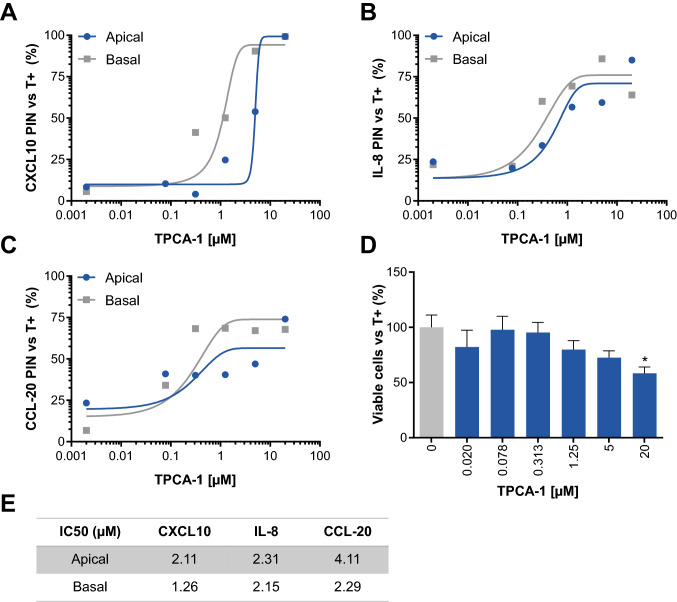
Exposure to TPCA-1 decreases inflammatory cytokine production by IEC. (**A–C**) Apical and basal percentage of inhibition (PIN) of CXCL10 (**A**) IL-8 (**B**) and CCL-20 (**C**) after TPCA-1 treatment. IEC from HIO donor 1 were grown under microfluidic conditions for 6 days before being exposed to TPCA-1 at the indicated concentrations. After 2 h of TPCA-1 exposure, cells were triggered for 24 h (LPS 100 ng/mL, IFN-γ 20 ng/mL) in the continuous presence of TPCA-1. Dots represent the PIN mean normalized to triggered but TPCA-1 untreated IEC (T +). The line depicts a non-linear regression between [TPCA-1] and cytokine secretion (n = 4–5). (**D**) Viability of IEC after 72 h of TPCA-1 exposure. Bars represent the percentage of viable cells average ± SEM with one-way ANOVA with Dunnett’s post-hoc test compared to triggered but TPCA-1 untreated tubules (T + ; n = 4–5). (**E**) Calculated CXCL10, IL-8 and CCL-20 IC50 values (µM) after 24 h of TPCA-1 exposure.

In an attempt to tone down the inflammation and assess the potential of the gut-on-a-chip model for drug discovery, we used TPCA-1, a known anti-inflammatory compound (Table 3). TPCA-1 is a selective inhibitor of human IκB kinase-2 (IKK-2) that inhibits production of pro-inflammatory cytokines in vitro and in vivo and inhibits NF-κB nuclear localization^19,36,37^. Apical and basal production of CXCL10, IL-8 and CCL-20 were efficiently inhibited in a dose-dependent manner in IEC (Fig. 5A–C). At IC50 concentrations (Fig. 5E), viability was not significantly altered and only the highest concentration of 20 µM significantly decreased viability (Fig. 5D). Interestingly, TPCA-1 always decreased basal production of cytokines more efficiently than their apical production (Fig. 5E). These results demonstrate that the gut-on-a-chip model could be a suitable alternative to standard organoid technology, which is of limited use in compound evaluation due to its inside-out configuration.

**Table 3:** Compound used in the study.

Generic name	CAS	Concentration	Diluent	Supplier
TPCA-1	507475-17-4	10 mM	DMSO	Sigma Aldrich (Saint Louis, MO, USA)

### Addition of a cellular immunological component

**Figure 6:**
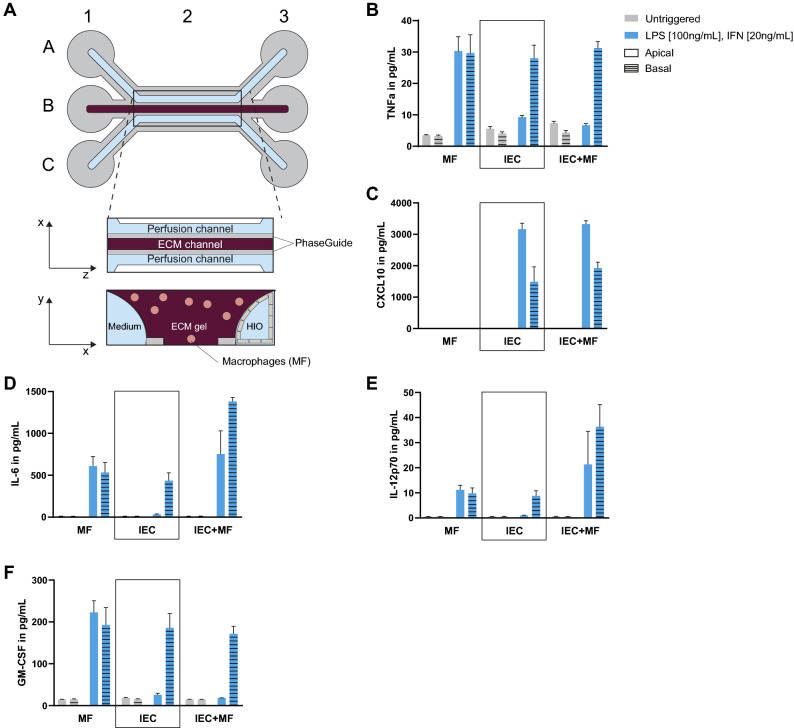
Induction of inflammatory conditions increases cytokine secretion of macrophages and IEC. (**A**) Schematic representation of the microfluidic co-culture modified from Fig. 1A. Macrophages are directly embedded in the ECM gel on which the epithelial tubule lay against. PhaseGuides allow a barrier-free culture. Illustration created with Adobe Illustrator software (version22.1, https://adobe.com/products/illustrator). (**B**–**F)** Apical and basolateral secretion of TNF-α (**B**), CXCL10 (**C**), IL-6 (**D**), IL-12p70 (**E**) and GM-CSF (**F**) in macrophages (MF), IEC from HIO donor 1 and the co-culture of both at Day 8 after a 24 h trigger (LPS 100 ng/mL, IFN-γ 20 ng/mL). Bars represent average cytokine production [pg/mL] ± SEM (n = 3–5).

As previously reported, M1 macrophages are over-represented and activated in the *lamina propria* of IBD patients and have been shown to contribute to IBD physiopathology^4,5^. To mimic this key feature of IBD, monocyte-derived macrophages were embedded in the ECM gel of our gut-on-a-chip and were allowed to directly interact with the intestinal epithelium due to the absence of physical barrier between the microfluidics channels (Fig. 6A). Once the IEC formed complete epithelial tubules, the co-culture was triggered with LPS and IFN-γ in both apical and basal channels, allowing the macrophages to polarize into M1 inflammatory macrophages, as demonstrated by an increase in TNF-α and IL-6 secretion (Fig. 6).

In order to characterize the inflammatory state of our model in more detail, we assessed the effect of the trigger on cytokine production by the epithelium and macrophages individually, as well as on both cell types. The apical and basal production of TNF-α, CXCL10, IL-6, IL-12p70, GM-CSF and IFN-γ were assessed for each mono-culture and the co-culture (Fig. 6B–F). From the cytokines analysed, only CXCL10 production was not upregulated by macrophages after trigger (Fig. 6C). In IEC, production of TNF-α, IL-6, IL-12p70 and GM-CSF were upregulated only on the basolateral side upon trigger (Fig. 6B,D-F), while CXCL10 production was upregulated in both the apical and basolateral compartments (Fig. 6C). After triggering the co-culture, production of TNF-α and GM-CSF were increased only at the basolateral side (Fig. 6B,F), whereas both the apical and basolateral production of CXCL10, IL-6 and IL-12p70 were increased (Fig. 6C-E). Interestingly, production of IL-12p70 and IL-6 were synergized in the co-culture (Fig. 6D,E). On the contrary, IEC showed an immunoregulatory effect on the apical production of TNF-α and GM-CSF production by macrophages in the co-culture (Fig. 6B,F).

To support the relevance of these findings, we investigated the mRNA expression of all analytes in colonic mucosal biopsy samples of control and IBD patients from previously published datasets^38^. The expression of all analytes, except *IL12A, IL12B* and *CSF2*, were found to be upregulated in the mucosa of patients with active UC or CD (Fig. S7). Overall, our results show that this trigger mixture could successfully induce an inflammatory state in the co-culture of HIO and macrophages, resulting in the upregulation of relevant epithelial and immune cytokines.

## Discussion

We report here the development of an in vitro model of the human intestine by integrating the organ-on-a-chip technology with organoid-based methods for culture of primary IEC from colorectal biopsies. A few gut-on-a-chip models integrating biopsy-derived or iPSC-derived IEC have been published recently^24,25,39,40^. However, none of them has addressed the importance of the immune component nor its implication to the study of IBD. Our gut-on-a-chip model differs from previous models by not only integrating primary human macrophages together with IEC, but also by inducing an inflammatory state of the epithelium similar to the one observed in IBD patients.

Firstly, we confirmed that all differentiated cell types present in the HIO were also represented in the gut-on-a-chip model. Upon microfluidic culture, the mRNA expression of markers associated with enterocytes and goblet cell differentiated cell types of the intestinal epithelium, as well as transcription factors favouring differentiation were increased, while markers for proliferation were decreased. Interestingly, stem cell marker *LGR5* expression was decreased by fluid flow as observed by previous studies^24^, while *OLFM4* expression was increased. Recent studies have demonstrated that OLFM4 was expressed at very low levels in the foetal intestine and in HIO cultures, whereas it was expressed at higher levels in the adult intestine^41,42^. It is unclear how a reduction in LGR5 positive cells might be linked to this observation and more investigation is necessary, it could however suggest that our gut-on-a-chip model has reached a higher level of maturity over static HIO culture.

Furthermore, IEC grown as a monolayer on an ECM gel under flow have higher expression of genes with a role in important intestinal functions such as digestion, intestinal transport and hormonal regulation over static HIO spheroids. This suggests that microfluidic gut-on-a-chip models might more adequately reflect organ-level functions over conventional organoids. Comparison of the expression level of a set of genes involved in colonic identity between microfluidic samples and in vivo intestinal segments revealed that there is no significant impact of microfluidic culture on intestinal phenotype. HIO donors, whether cultured in static or microfluidic conditions, highly resembled the human colonic mucosa. The differences between the general intensities of the signals could be explained by the different technologies used. Despite this difference in signal intensity, the relative gene expression profile was decidedly similar to that of genes in the human colon, making our model particularly appropriate for UC modelling, which mainly affects that area of the gastrointestinal tract. Previously published Transwell and gut-on-a-chip Caco-2 models, on the other hand, more closely resembled the human small intestine. One should therefore be careful using Caco-2 cells to study colon-specific mechanisms and pathologies such as UC, as our results suggest that they strictly represent cells from the small intestine, despite the fact that they have been described to express both enterocytes and colonocytes markers^10^. Furthermore, in both the HIO and Caco-2 case, the microfluidic culture did not significantly alter the expression of genes involved in intestinal phenotype. Despite the lack of regulation of RNA expression profiles of this specific set of genes, other genes involved in intestinal transport and metabolism were highly represented by microfluidic culture. Furthermore, other studies have also shown that HIO gut-on-a-chip models led to increased intestinal functions compared to organoids or Caco-2 gut-on-a-chip models^24,39^. Thus, for assessments such as drug metabolism and response to nutrients, microfluidic models might be more informative of the in vivo situation.

In this study, we have used the original HISC medium recipe as originally published by Hans Clevers group presented at Table 2^28^. To optimize IEC attachment in the microfluidic plate, ALK5 inhibitor A83-01 was withdrawn for the first 48 h, as previous studies have also done^43^. When withdrawn for 48 h, we did not obverse any upregulation of EMT markers. However, it cannot be excluded that the removal of A83-01 could be responsible for some molecular changes observed in our RNA-sequencing analyses. However, we did not observe an upregulation of pathways regulated by TGF-β such as EMT, proliferation or apoptosis. We have, in fact, observed a clear reduction in proliferation markers expression.

We next inquired whether the gut-on-a-chip could be applied to model inflammatory responses. To do so, we applied a mixed trigger composed of bacterial LPS and cytokine IFN-γ to the microfluidic model. We first confirmed the induction of an inflammatory state by RNA-sequencing of HIO. Pathways involved in cytokine regulation and bacterial response that are dysregulated in IBD patients were found to be overrepresented in triggered IEC, confirming the general inflammatory state of the model and supporting its physiological relevance.

Naturally, IBD is a multi-factorial disease that cannot be solely recapitulated by one cell type. Macrophages, which were shown to be overrepresented and activated in IBD^4,5^, were therefore included into our model to increase its complexity. Upon trigger, macrophages were activated into pro-inflammatory M1 macrophages and secreted immune-relevant cytokines TNF-α and IL-6. The inflammatory trigger also activated IEC, which in turn, secreted epithelial cytokines. This was particularly the case for IL-12p70 and IL-6 whose secretion was synergized in the co-culture. Contrarily, IEC showed an immunoregulatory effect on the apical production of TNF-α and GM-CSF production by macrophages in the co-culture. IEC are capable of secreting modulators that can modulate the cytokine secretion of macrophages, such as cholecystokinin and glucagon-like peptide (GLP) 1 and 2^44,45^. Particularly, IEC have been reported to induce differentiation of normal macrophage into a tolerogenic phenotype resembling intestinal macrophages^46^. In all cases, the basolateral production of inflammatory cytokines upon trigger was always greater than the apical production, suggesting that IEC respond to an inflammatory state by secreting analytes at the basolateral side. The reason for this could be that IEC are involved in recruitment of immune effectors residing in the underlying *lamina propria* or circulating in the blood through the secretion of chemokines and cytokines at the basolateral site^47^.

Our study did not assess whether other changes in the medium composition would further affect IEC differentiation and the cellular responses engaged. Research groups have now shown that innate immune responses of IEC are modulated by the removal of Wnt3a conditioned medium, which alters the state of differentiation of IEC. For instance, removal of Wnt3a was shown to induce MHC II expression which can in turn promote TLR-triggered innate immune responses^48^. Similarly, the presence of anti-oxidants in the HISC medium have also been shown to influence innate immune responses of IEC^49^. Notably, jejunal organoids cultured in medium devoid of antioxidants were more responsive to host and microbial inflammatory signals. While we demonstrate that innate immune responses were successfully induced in our model, it would become important in the future to assess whether the modification of the HISC medium composition alters those responses in IEC and whether the IEC-immune cell relationship is consequently affected. Furthermore, our study focused on the effect of microfluidic culture and pro-inflammatory stimulation on the intestinal epithelium, but we did not assess the contribution of individual cell types to the phenotype. It would be of particular interest to investigate cellular changes occurring in goblet and Paneth cells during co-culture with macrophages, as abnormal cell function has been described in IBD patients^50,51^.

One of the drawbacks of the organoid methodology is their enclosed lumen, making drug exposure studies difficult. In addition, it does not allow to simultaneously measure the secretion of analytes at both the apical and basolateral site. The novel gut-on-a-chip model presented in this study transcends these limitations and allowed IEC exposure to TPCA-1 compound in a polarized manner. TPCA-1, by selectively binding the ATP pocket of IKK-2, prevents the phosphorylation of inhibitor of NF-κB (IκB), preventing NF-κB nuclear translocation and the activation of genes involved in inflammation and other immune responses^36^. TPCA-1 markedly reduced the epithelial production of CXCL10, IL-8 and CCL-20 by IEC. The compound has previously been shown to reduce IL-8 by HT-29 cells^52^ as well as CXCL10, IL-8 and CCL-20 production by Caco-2 cells^19^. Furthermore, NF-κB has been shown to be activated in mucosal biopsies of patients with active IBD and the use of steroids, by decreasing NF-κB activity, have shown to reduce clinical symptoms of patients^53^. Hence, our proof-of-concept experiment show preliminary evidence that our model could potentially be used to assess the efficacy of drug candidates on cytokine secretion in the intestine. Importantly, our model offers numerous advantages over Caco-2 models currently used in drug absorption studies^54,55^. Indeed, although Caco-2 cells have been found to express a large number of enzymes and transporters present in the normal human intestinal epithelium, studies suggest that there are variations between gene expression profiles of transformed epithelial cell lines like Caco-2 and the normal human intestinal epithelium^56,57^. This could, in turn affect the accuracy and relevance of results obtained in this system. Furthermore, while some studies show the limited presence of MUC2 expression in Caco-2 cells under specific conditions^21^, they are overall considered enterocyte-like only and do not reflect the diversity of the intestinal epithelium. By using human primary IEC derived from biopsies, we addressed the limitations incurred by the use of Caco-2 cells. In addition, culturing the IEC in microfluidic conditions corrected the inside-out configuration of HIO and increased the expression of intestinal transporters such as SLC superfamily members that are known to be important for the transport of drugs through the epithelium^58^.

Our novel primary human gut-on-a-chip model could therefore not only be used to study drug transport, absorption and toxicity but could also be potentially of use in studying intestinal development, tissue-tissue interactions, host–pathogen connections as well as regenerative medicine. In the future, the implementation of other cell components participating in IBD pathogenesis, such as supplementary immune cell types to reflect the heterogeneity of the mucosal immune system, would allow us to reflect in vivo physiology more closely. Similarly, access to IBD-patient material could allow us to investigate patient-specific disease mechanisms and therapy response, opening the doors to personalized medicine. The advent of the organ-on-a-chip technology allows us to separately control physical and mechanical factors (e.g., fluid flow) as well as cellular components to better understand and model intestinal homeostasis and diseases of the gastrointestinal tract such as IBD.

## Methods

### Ethics statement

The research described here has been performed according to applicable Dutch national ethics regulations and was conducted within Galapagos BV (Leiden, NL). The use of human cells and associated protocols were approved by the Galapagos biobanking committee. Scientists from Galapagos BV are qualified to perform research using human material and have appropriate facilities and equipment available to comply with applicable laws, regulations and internal rules related to handling and storage of the material. The human material was obtained from Sanquin (Amsterdam, NL), Tissue Solutions (Glasgow, UK) or the Hubrecht Institute (Utrecht, NL). The suppliers have confirmed to Galapagos BV that informed consent from the donors to use the material for research purposes was received. The cells were solely used for target and drug discovery and were not used for human experimentation or therapy. All material is and will remain anonymized.

### In vitro culture of human intestinal organoids

Intestinal crypts were isolated from intestinal biopsies as previously described^15,28^, resuspended in Matrigel (Corning) and polymerized at 37 °C. Human intestinal organoids (HIO) were grown in Human intestinal stem cell (HISC) medium^15,28^, passaged at a ratio of 1:10 to 1:12 every 10–14 days and used between passages 4 and 15. The final composition of the HISC medium is shown at Table 2. HISC medium was produced in Advanced DMEM/F-12 (Gibco, #12634) supplemented with 1%v/v Glutamax (Gibco, #35050), 1%v/v HEPES (Gibco, #15630) and 1%v/v Pen/Strep (Gibco, #15140). 2X-concentrated HISC medium was diluted in a 1:1 ratio in Wnt3a conditioned medium. To produce Wnt3a conditioned medium, L Wnt3a cells (CRL-2647) were grown in Advanced DMEM/F-12 (Gibco, #12634) supplemented with 10% FBS (Bovogen, #SFBS), 1%v/v Glutamax (Gibco, #35050), 1%v/v HEPES (Gibco, #15630) and 1%v/v Pen/Strep (Gibco, #15140) in presence of 125 µg/mL Zeocin (Thermo Fisher, #R25001).

### Isolation and in vitro culture of human macrophages

Human peripheral blood mononuclear cells (PBMCs) were isolated from buffy coats using density gradient centrifugation on Ficoll-Paque according to manufacturer protocol. Monocytes were positively isolated using magnetic CD14 microbeads (Miltenyi, #130-050-201) and were cultured in a low-attachment flask for 5 days in RPMI (Gibco, #72400) supplemented with heat-inactivated FBS (Gibco, #F8067), Pen/Strep (Gibco, #15140) and [100 ng/mL] rh M-CSF (ImmunoTools, #11343115) to differentiate towards macrophages.

### Gut-on-a-chip coating, seeding and culture

#### Coating of gut-on-a-chip without macrophages

An ECM gel was prepared by mixing 1 M HEPES (Gibco, #15630), 37 g/L NaHCO_3_ pH9.5 (Sigma Aldrich, #S5761) and rat-tail Collagen I (Cultrex, #3447–020-01) at a 1:1:8 v/v/v ratio (HEPES/NaHCO_3_/Collagen I). A 1.6 µL volume was dispensed in the middle channel of a 3-lane 400 μm OrganoPlate (Mimetas, #4003-400-B) and let to polymerize for 15 min in a 37 °C environment in presence of 5%CO_2_.

#### Coating of gut-on-a-chip with macrophages

Macrophages were harvested, pelleted and resuspended in the ECM gel at a concentration of 1.1 × 10^6^ cells/mL. Embedded macrophages were seeded in the middle lane of a 3-lane 400 μm OrganoPlate (Mimetas, #4003-400-B) in a 1.8 µL volume, thus making 20,000 macrophages/chip. Coated plates were placed in a 37 °C incubator supplemented with 5% CO_2_ for 15 min until the ECM solidified.

#### Gut-on-a-chip seeding

During ECM polymerization, HIO were retrieved from Matrigel droplets and made single cells using TryplExpress (Gibco, #12,605) and mechanical dissociation as previously reported^15,28^. 100,000 HIO cells were seeded in a 2 µL volume in the upper channel of the OrganoPlate and left to adhere against the gel for 45 min. After incubation, 50 µL of HISC medium devoid of A83-01 (attachment medium) was added to the upper inlet and the plates were returned to the incubator at a 70° angle for 4–6 supplementary hours. After 4–6 h, 50 µL of attachment medium was added to all remaining inlets and outlets and the plates were placed back in the incubator without medium perfusion for 24 h. On Day 1, plates were placed on an interval rocker (Perfusion Rocker Mini, Mimetas) switching between a + 7° and − 7° inclination every 8 min (37 °C, 5% CO_2_) leading to a 121.2 µl/hr perfusion rate^59^. Attachment medium was replaced with HISC medium two days after seeding, and HISC medium was refreshed every three days after that. During all experimentation, the lower channel medium was supplemented with [100 ng/mL] rh M-CSF (ImmunoTools, #11,343,115) to maintain the macrophages differentiated.

#### Gut-on-a-chip activation

6 days after seeding, the co-culture was activated using [100 ng/mL] LPS (Sigma, #L3024) and [20 ng/mL] rh IFN-γ (ImmunoTools, #11343534) in HISC medium in both upper and lower channels for 16–24 h. Effluents were collected from apical (upper channel) and basolateral (lower channel) compartments at specified times and kept at − 20 °C until downstream analyses were performed.

#### Gut-on-a-chip exposure to compound

6 days after seeding, the co-culture was pre-exposed to compound concentrations ranging from 0.02 µM to 20 µM in 0.22% DMSO in upper and lower channels for 2 h. After 2 h, the trigger (100 ng/mL LPS, 20 ng/mL IFN-γ) in presence of 0.02–20 µM compound was applied in upper and lower channels. Effluents were collected from apical and basal channels 24 h and 72 h after compound exposure and viability was assessed 72 h after compound exposure.

### Assessment of cytokine production

The concentration of Granulocyte–Macrophage Colony-Stimulating Factor (GM-CSF), Interleukin-12 unit p70 (IL-12p70), Interleukin-6 (IL-6), Interleukin-8 (IL-8/CXCL8), IFN-Gamma-Inducible Protein-10 (IP-10/CXCL10), Macrophage Inflammatory Protein-3 (CCL-20/MIP3A) and Tumor Necrosis Factor-Alpha (TNF-α) in collected effluents was determined by a human multiplex assay (Thermo Fischer) on a Luminex FlexMap 3D (Merck Millipore) according to manufacturer protocol.

### Assessment of viability

72 h after compound treatment, WST-8 reagent (Sigma Aldrich) was mixed in HISC medium in a 1:10 ratio and 25µL was added to upper and lower inlets and outlets. Plates were incubated at 37 °C for 4 h and absorbance signals were measured (450 nm monochromator) with a multi‐well plate fluorimeter (Envision, Perkin Elmer).

### Immunocytochemistry

Cells were fixed and prepared for immunohistochemistry as previously described^19,29^. The primary antibodies used were anti-E-CADHERIN (Abcam #AB1416, 1:100), anti-EZRIN (Invitrogen #MA5-13,862, 1:50), anti-MUC2 (Invitrogen #MA5-12,345, 1:50). Secondary antibody used was donkey anti-mouse AlexaFluor647 (Molecular Probes #A31571, 1:250) in combination with ActinGreen 488 ReadyProbes Reagent (Thermo Fisher, #R37110) and NucBlue Fixed Cell ReadyProbes Reagent (Thermo Fisher, #R37606) for 1 h at room temperature as explained previously^19^. Images were acquired on the InCell 6000 (GE Healthcare Life Sciences).

### RNA isolation

RNA was isolated from the microfluidic chips using TRI-reagent (Sigma Aldrich). In a nutshell, 100 µL of TRI-reagent was added to the top inlet and 25 µL to the top outlet of the OrganoPlate. The plate was placed under an angle for 15 min. For standard HIO cultures, HIO were harvested from Matrigel droplets using cold PBS (Gibco), pelleted and resuspended in TRI-reagent. The lysed cells were harvested and a phenol/chloroform extraction was performed according to manufacturer’s instructions. Isolated RNA was quantified on the Nanodrop 2000 (Thermo Fisher), was treated with amplification grade DNAse I (Invitrogen) for 15 min at room temperature and inactivated with incubation with EDTA.

### Reverse transcription and qRT-PCR

RNA quality control was performed using the 2100 Bioanalyzer microfluidic gel electrophoresis system (Agilent). Removal of gDNA contamination was performed using the Heat&Run gDNA removal kit (ArticZymes). cDNA synthesis was performed using the iScript Advanced cDNA Synthesis Kit (Bio-Rad) with 600 ng of total RNA input. Each qPCR reaction used 5 ng cDNA equivalents of RNA as input and a SYBR Green I mastermix (Bio-Rad) in a total volume of 5µL. All qPCR reactions were run in duplicate in 384-well plates on a CFX384 instrument (Bio-Rad). All data presented was normalized to housekeeping genes *RPS18*, *HPRT1*, *GUSB* and *YWHAZ* (Table 4).

**Table 4:** Probes used in the study.

Gene	Ensembl ID	PrimePCR assay ID
*ALPI*	ENSG00000163295	qHsaCED0001947
*CDH1*	ENSG00000039068	qHsaCID0015365
*CDX2*	ENSG00000165556	qHsaCID0013185
*CHGA*	ENSG00000100604	qHsaCED0003225
*EZR*	ENSG00000092820	qHsaCID0016058
*GUSB*	ENSG00000169919	31189388
*HES1*	ENSG00000114315	qHsaCED0006922
*HPRT1*	ENSG00000165704	31189301
*KLF4*	ENSG00000136826	qHsaCED0044721
*LGR5*	ENSG00000139292	qHsaCED0034618
*LYZ*	ENSG00000090382	qHsaCID0008160
*MKI67*	ENSG00000148773	qHsaCID0011882
*MUC2*	ENSG00000198788	qHsaCID0011696
*OCLN*	ENSG00000197822	qHsaCED0038290
*OLFM4*	ENSG00000102837	qHsaCID0021022
*PCNA*	ENSG00000132646	qHsaCID0012792
*RPS18*	ENSG00000231500	31189344
*SI*	ENSG00000090402	qHsaCID0008471
*TJP1*	ENSG00000104067	qHsaCID0018062
*VIL1*	ENSG00000127831	qHsaCID0017926
*YWHAZ*	ENSG00000164924	31189446

### RNA-sequencing

Library preparations, sequencing reactions were conducted at GENEWIZ, LLC. (South Plainfield, NJ, USA) and have been described before^60,61^. RNA samples were quantified using Qubit 2.0 Fluorometer (Life Technologies, Carlsbad, CA, USA) and RNA integrity was checked with 4200 TapeStation (Agilent Technologies, Palo Alto, CA, USA). Ribosomal RNA depletion was performed using Ribozero rRNA Removal Kit (Illumina, San Diego, CA, USA). RNA sequencing library preparation used NEBNext Ultra RNA Library Prep Kit for Illumina by following the manufacturer’s recommendations (NEB, Ipswich, MA, USA). Briefly, enriched RNAs were fragmented for 15 min at 94 °C. First strand and second strand cDNA were subsequently synthesized. cDNA fragments were end repaired and adenylated at 3′ends, and universal adapter was ligated to cDNA fragments, followed by index addition and library enrichment with limited cycle PCR. Sequencing libraries were validated using the Agilent Tapestation 4200 (Agilent Technologies, Palo Alto, CA, USA), and quantified by using Qubit 2.0 Fluorometer (Invitrogen, Carlsbad, CA) as well as by quantitative PCR (Applied Biosystems, Carlsbad, CA, USA). The sequencing libraries were multiplexed and clustered on one lane of a flowcell and loaded on the Illumina HiSeq instrument according to manufacturer’s instructions. The samples were sequenced using a HiSeq 2 × 150 Paired End configuration. Image analysis and base calling were conducted by the HiSeq Control Software. Raw sequence data (.bcl files) generated from Illumina HiSeq was converted into fastq files and de-multiplexed using Illumina's bcl2fastq 2.17 software. One mis-match was allowed for index sequence identification.

### Gene expression quantification and differential gene expression analysis

Quality reports for raw reads were generated using FastQC toolkit, followed by Trimmomatic trimming of raw reads using the following parameters (adapters: TruSeq3-PE.fa; sliding window:4; leading quality threshold:3). Obtained reads after trimming were checked for quality with FastQC and MultiQC toolkits and were mapped against the human genome (GRCh38.98) using STAR^62^ and Kallisto^63^. Gene counts from each sample were used for subsequent analyses; STAR generated counts were investigated using DESeq2 package^64^ and Kallisto – with Sleuth package^65^; this strategy was chosen to specifically compare for mapping accuracy and potential batch effects. DESeq2 was subsequently used to estimate variance-mean dependence in generated count data, followed by variance stabilizing transformation to test for differential expression using a model based on the negative binomial distribution. Fold changes and adjusted p-values (q-value) for sample comparison were calculated setting at alpha = 0.05 and pAdjustMethod = “BH”. Differentially expressed genes (q-value < 0.05) were analysed based on log2fold change as well as rlog transformed variability across different conditions. Heatmaps were built selecting distance function: “euclidean” and hierarchical clustering function: “complete”. Statistical analysis and visualization of functional profiles for genes was performed with Clusterprofile package based on GO and KEGG datasets^66^. All analyses performed with R for statistical programming (version 3.5.3) on the RStudio IDE (Version 1.2.5019). Additional datasets used for comparison: GSE109471^24^ (Caco-2 Transwell, Caco-2 gut-on-a-chip, duodenum-on-a-chip, duodenum organoid samples), GSE14938^30^ (human normal duodenum/jejunum/ileum samples), GSE9254^31^ (human normal colon samples). All additional samples were analysed and normalized based on the platform used for read generation integrating into existing processing pipeline.

“MetaCore from Clarivate Analytics” was used to parse multiple disease and metabolic pathways against the gene set of interest (selecting upregulated genes above the threshold p < 0.05). Pathways were generated using the following parameters (network building: direct interactions, species: human, selecting for binding and functional interactions).

### Investigation of inflammatory cytokine expression in IBD patients

Gene expression across patients samples was examined in curated databases using Genevestigator^67^. Dataset GSE59071 allowed the investigation of gene expression in the colonic mucosa of healthy and IBD patients^38^.

### Statistics

Data analysis was performed with GraphPad Prism software version 8 (GraphPad Software, La Jolla, CA, USA). All values are expressed as mean ± standard error of the mean (SEM), unless indicated otherwise. A two-tailed, unpaired Student’s t-test was used to determine the statistical significance when two groups of data were analysed. When more than two groups were analyzed, parametrical ANOVA was used. Differences with p values ≤ 0.05 were considered significant (ns p > 0.05, * p ≤ 0.05, ** p ≤ 0.01, *** p ≤ 0.001, **** p ≤ 0.0001). Results shown are from one representative experiment containing at least three technical replicates. The number of technical replicates is presented in the legend of each figure.

## Supplementary Information

Supplementary Information 1.

Supplementary Information 2.

Supplementary Information 3.

## Data Availability

The datasets generated during and/or analysed during the current study are available in the GEO repository, [GSE153191].

## Abbreviations

CCL-20: C–C Motif Chemokine Ligand 20
CD: Crohn’s disease
CML: Chronic myeloid leukemia
CXCL10: C-X-C motif chemokine 10
ECM: Extracellular matrix
EMT: Epithelial-mesenchyme transition
FDR: False discovery rate
HIO: Human intestinal organoids
HISC: Human intestinal stem cell
IBD: Inflammatory bowel disease
IEC: Intestinal epithelial cells
IFN-γ: Interferon gamma
IL-8: Interleukin-8
iPSC: Induced pluripotent stem cells
LPS: Lipopolysaccharide
M-CSF: Macrophage stimulating factor
PDMS: Polydimethylsiloxane
PIN: Percentage of inhibition
rh: Recombinant human
SLC: Solute carrier
UC: Ulcerative colitis
