# Supplementary material for: Development of a human primary gut-on-a-chip to model inflammatory processes
Source: Sci Rep. 2020 Dec 8;10:21475. doi: 10.1038/s41598-020-78359-2 (PMC7722760; doi:10.1038/s41598-020-78359-2)
Supplement: Supplementary file 1 — Supplementary Information 1. [file 41598_2020_78359_MOESM1_ESM.docx]

# TITLE: DEVELOPMENT OF A HUMAN PRIMARY GUT-ON-A-CHIP TO MODEL INFLAMMATORY PROCESSES

# AUTHORS:

Claudia Beaurivage^1,2^, Auste Kanapeckaite^1^, Cindy Loomans^1^, Kai S. Erdmann^2^, Jan Stallen^1^, Richard A.J. Janssen^1,*^

^1^Galapagos BV, Leiden, South Holland, 2333CL, The Netherlands

^2^Department of Biomedical Science, Faculty of Science, University of Sheffield, Sheffield, South Yorkshire, S10 2TN, United Kingdom

* Corresponding author

# SUPPLEMENTARY INFORMATION

**
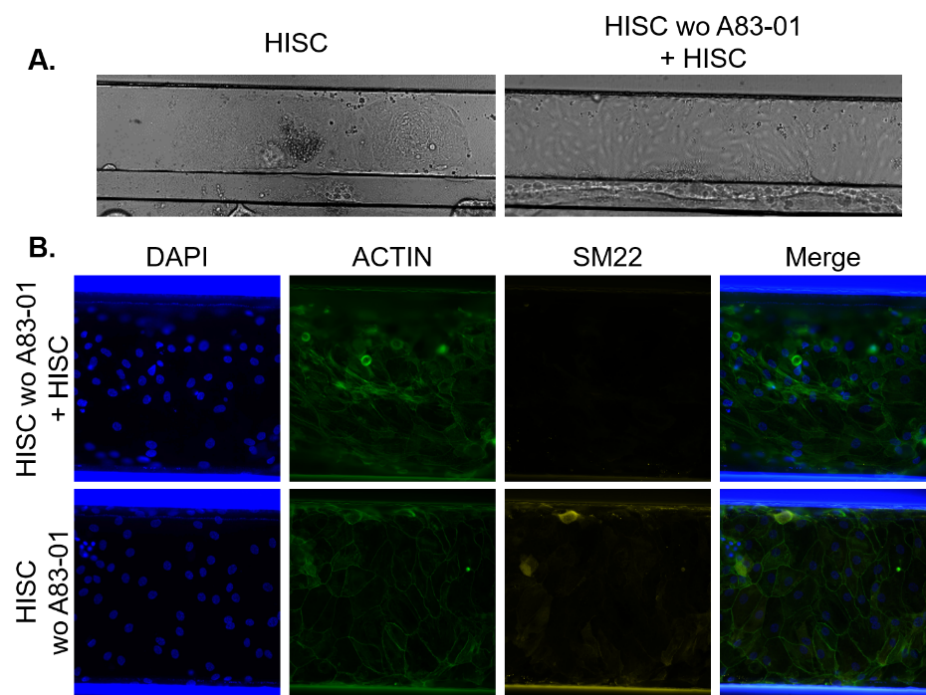
**

**Figure S1. Optimized HISC medium favours IEC attachment**

**A.** Representative 10X brightfield stitched images of HIO donor growing in microfluidic conditions for 8 days in HISC medium (left panel) or in HISC without A83-01 for 2 days followed by HISC for 6 days (right panel, wo=without). **B**. Representative 20X photographs of polarization marker ACTIN and EMT marker SM22 in HIO (donor 1) grown in microfluidic conditions for 8 days in HISC medium without A83-01 for 2 days followed by 6 days of HISC medium (top panel) or in HISC without A83-01 for 8 days (bottom panel). Blue depicts nuclei by DAPI staining. Images were created with the Image J software (version 1.47v, https://imagej.nih.gov/ij/).

**
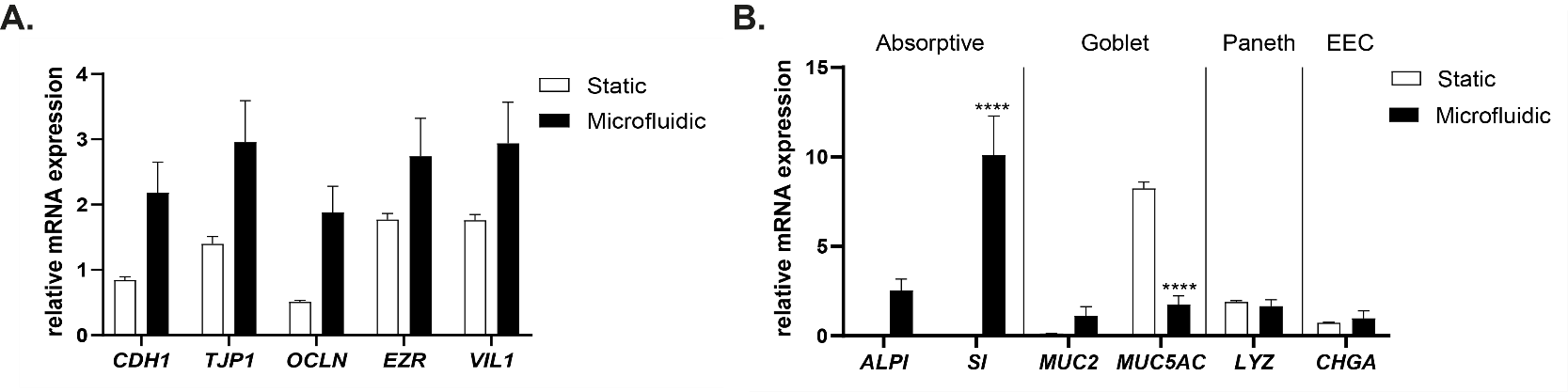
**

**Figure S2. HIO cultured under microfluidic conditions show higher expression of polarization and differentiation markers**

mRNA expression level of (**A.**) polarization markers *CDH1*, *TJP1*, *OCLN*, *EZR* and *VIL1* and (**B.**) differentiation markers *ALPI*, *SI*, *MUC2*, *MUC5AC*, *LYZ* and *CHGA* in HIO donor 3. HIO were grown in static condition or in monolayers subjected to fluid flow (microfluidic) for 8 days. Graph shows average relative expression values normalized to *RPS18*, *HPRT1*, *GUSB* and *YWHAZ* ± SEM with two-way ANOVA with Sidak’s post-hoc test compared to the static condition (n=3).

**
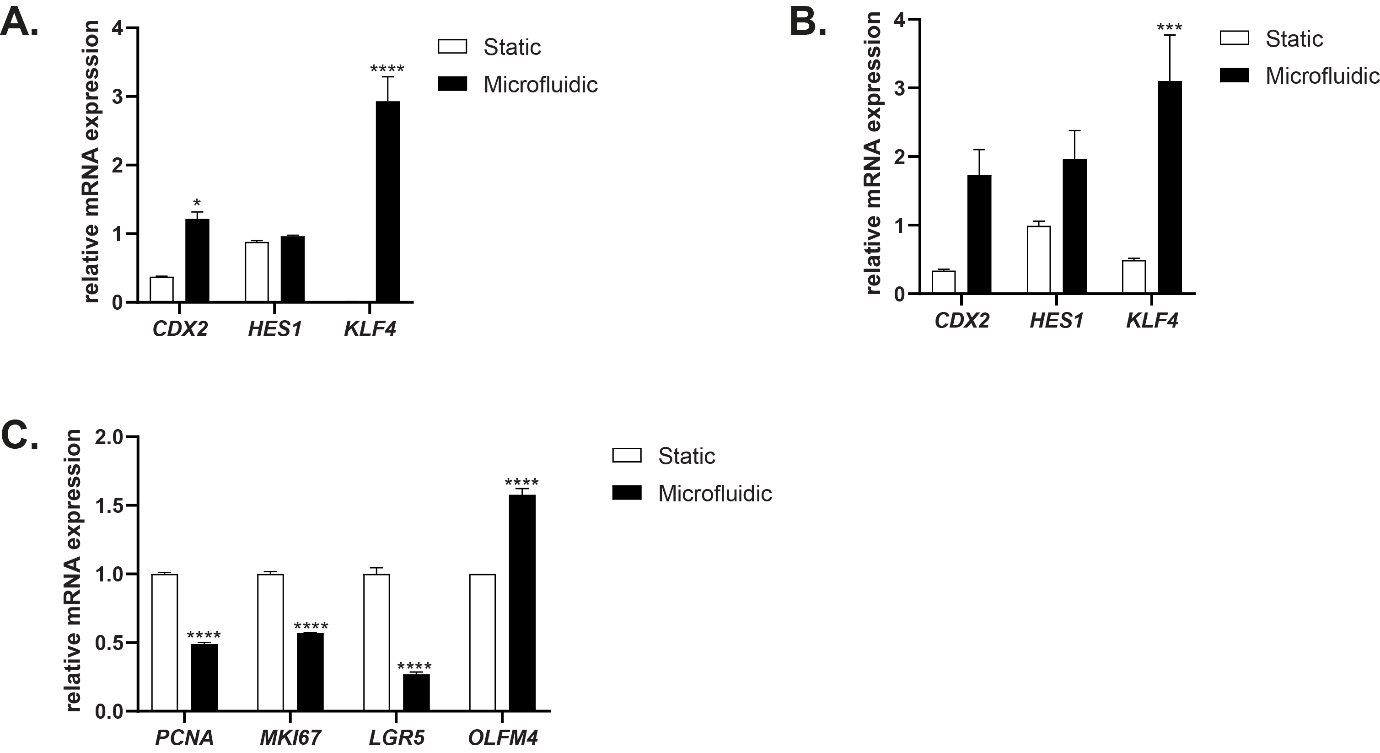
**

**Figure S3. HIO cultured under microfluidic conditions seem to favour differentiation over proliferation pathways**

**A-B.** mRNA expression level of differentiation transcription factors *CDX2*, *HES1* and *KLF4* in HIO donor 2 (**A.**) and HIO donor 3 (**B.**). **C.** mRNA expression level of proliferation markers *PCNA* and *MKI67* and stem cell markers *LGR5* and *OLFM4* in HIO donor 3. **A-C**. HIO were grown static condition or in monolayers subjected to fluid flow (microfluidic) for 8 days. Graph shows average relative expression values normalized to *RPS18*, *HPRT1*, *GUSB* and *YWHAZ* ± SEM with with two-way ANOVA with Sidak’s post-hoc test compared to the static condition (n=3).


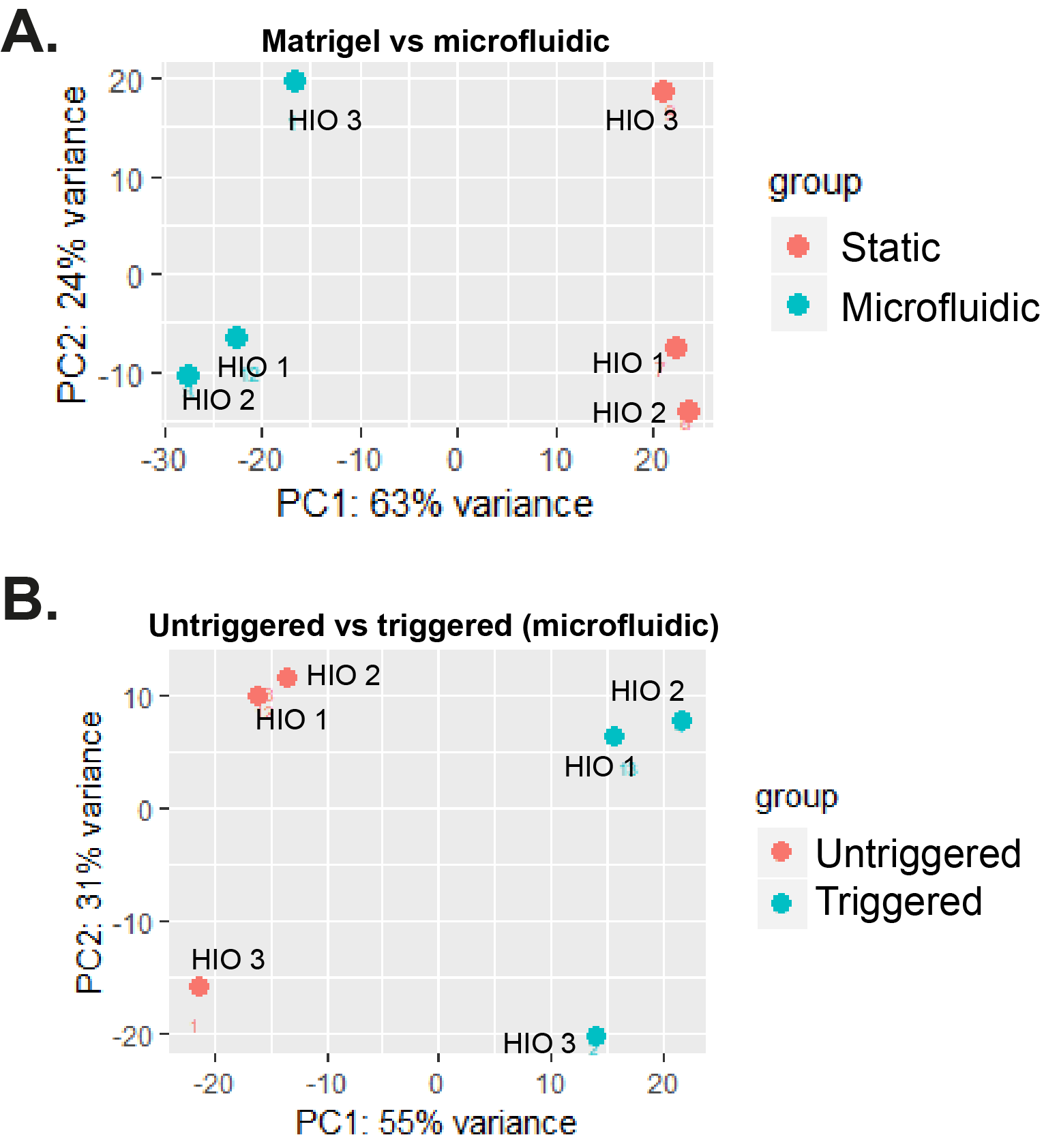


**Figure S4. Principal component analysis (PCA) plots**

**A.** 3 HIO donors were grown in static (red) or microfluidic conditions (blue). **B.** 3 HIO donors were grown in microfluidic conditions and triggered using LPS [100 ng/mL] and IFN-γ [20 ng/mL] for 24 h (blue) or left untriggered (red). In the 2 plots, a clear separation between the 2 experimental groups is observed. HIO donor 3 also shows a clear distinction, but is segregated from HIO donor 1 and 2 in all groups.

**
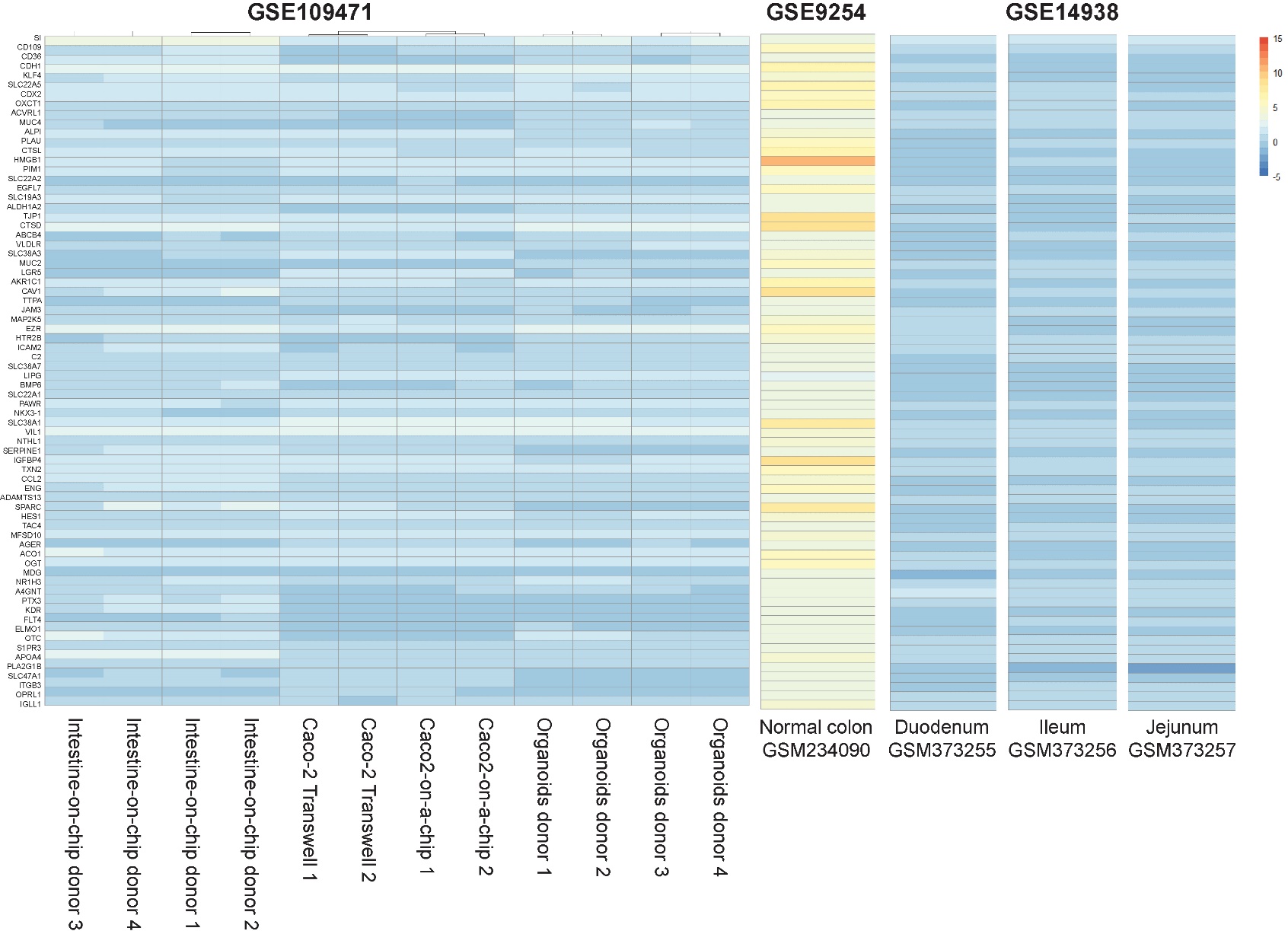
**

**Figure S5. RNA expression profiles of Caco-2 gut-on-a-chip resemble small intestinal segments**

Heatmap representation of the RNA expression profile of 72 genes defining intestinal identity in HIO from three donors grown in static or microfluidic conditions for 8 days, compared with human normal colon from study GSE9254 (LaPointe et al., 2008) and human normal small intestine sections (duodenum, jejunum, ileum) from study GSE14938 (She et al., 2009).


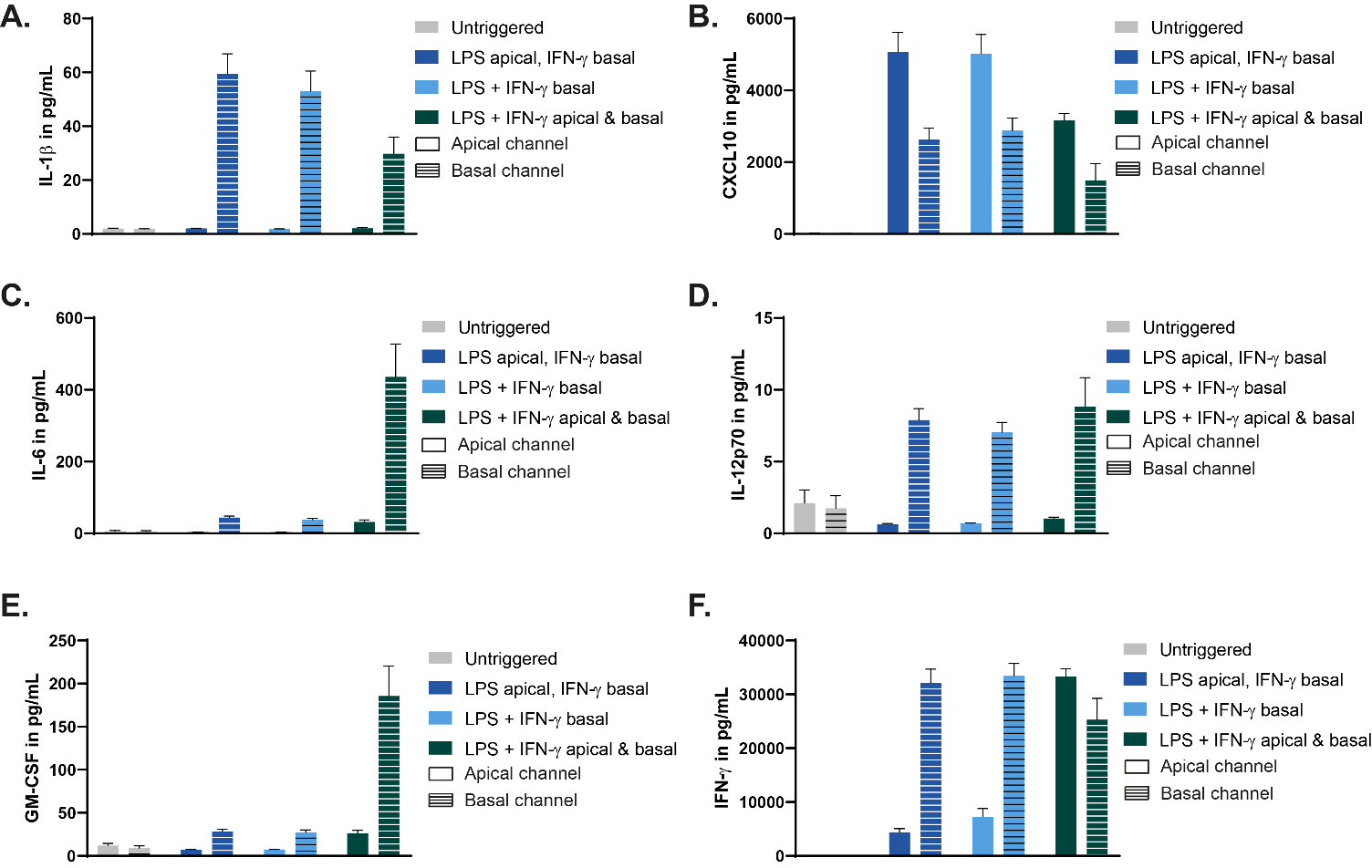


**Figure S6. Asymmetric responses of IEC to inflammatory stimuli**

**A-F**. Apical and basolateral secretion of IL-1β (**A.**), CXCL10 (**B.**), IL-6 (**C.**), IL-12p70 (**D.**), GM-CSF (**E.**) and IFN-γ (**F.**) in IEC from HIO donor 2 at Day 8 after application of LPS [100 ng/mL] and IFN-γ [20 ng/mL] for 24 h. Trigger constituents were either segregated in different channels (LPS apical, IFN- γ basal; dark blue bars), applied together in the basal channel only (pale blue bars) or applied together simultaneously in the apical and basal channels (green bars). Bars represent average cytokine production [pg/mL] ± SEM (n=4-10).

**
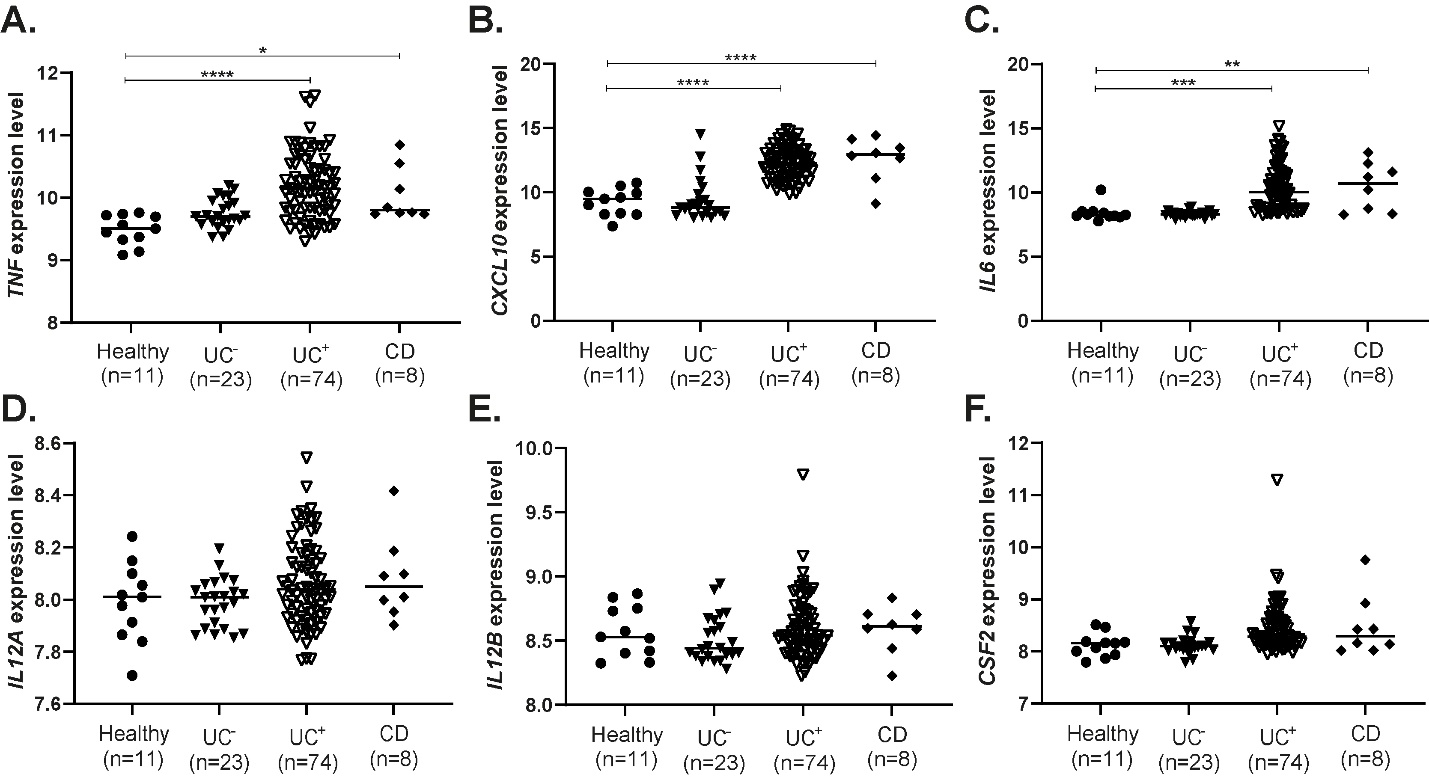
**

**Figure S7. The expression of multiple inflammatory cytokines is overexpressed in the colonic mucosa of IBD patients**

**A-G**. Gene expression of *TNF* (**A.**), *CXCL10* (**B.**), *IL6* (**C.**), *IL12A* (**D.**), *IL12B* (**E.**) and *CSF2* (**F.**) in mucosal colonic biopsies of human patients from GSE59071 (UC^-^= inactive ulcerative colitis, UC^+^=active ulcerative colitis, CD=Crohn’s disease) (Vanhove et al., 2015). Each symbol represents expression data from one individual patient, horizontal bars denote median values. One-way ANOVA with Dunnett’s post-hoc test compared to healthy samples (n=8-74).
